# Supplementary material for: Heavy metals in the Arctic: Distribution and enrichment of five metals in Alaskan soils
Source: PLoS One. 2020 Jun 3;15(6):e0233297. doi: 10.1371/journal.pone.0233297 (PMC7269202; doi:10.1371/journal.pone.0233297)
Supplement: Attachment — Submitted filename: Soil Alaskan Arctic 2019.docx [file pone.0233297.s004.docx]

The manuscript “Heavy metal in the Arctic” addresses important scientific problem and uses unique set of data which should be presented to the scientific community

However the treatment of the data, comparison with other available studies and discussion of mechanisms and consequences are not at the level suitable for an academic publication

**Major issues:**

Since the authors have the data for various depths, they should attempt to estimate the stock of metal sin frozen and seasonally thawed horizons

The comparison with other studies and with available health standards (L213-237) lacks statistical criteria of exceedance. Sometimes the average (or median) is higher than the health standards but the uncertainty (interquartile, Table 1) are not. The authors should also group the soils by a degree of exceedance (i.e., by a factor of 2, of 10.. etc)

The authors often use term “lower or “higher (ex. L 244) when comparing large number of values. The big data base should be compared using very different, and well developed today, statistical criteria. The whole discussion in L 240-255 thus seems to be not supported by rigorous statistical treatment.

Another example is L272-274. Such comparison of average values is not acceptable. The natural range of metal concentrations is at least 1 order of magnitude. Does this above mean > 10 times higher? If not, it is not necessarily ‘higher’, and serious statistical treatment is needed to judge the difference.

The PCA (and HCA) treatment of metal concentrations together with other soil properties may be useful to reveal controlling factors and correlations between elements (and other soil parameters)

L288-333: This text represents some review/perspectives of research, not directly linked to the obtained results. Part of this text can be in the Introduction, but the Discussion should be focused on results of this study and explanation of the pattern of metal distribution in soils with respect to landscape parameters, anthropogenic factors, rock lithology, permafrost distribution, soil type etc. Examples for the Scandinavian Arctic soils:

Reimann, C., de Caritat, P., 2005. Distinguishing between natural and anthropogenic

sources for elements in the environment: regional geochemical surveys versus

enrichment factors. Sci. Total Environ. 337, 91–107

Reimann, C., Banks, D., de Caritat, P., 2000a. Impacts of airborne contamination on

regional soil and water quality: the Kola Peninsula, Russia. Environ. Sci. Technol.

34, 2727–2732.

Reimann, C., Banks, D., Kashulina, G., 2000b. Processes influencing the chemical

composition of the O-horizon of podzols along a 500 km north-south profile

from the coast of the Barents Sea to the Arctic Circle. Geoderma 95, 113–139.

Reimann, C., Kashulina, G., de Caritat, P., Niskavaara, H., 2001. Multi-element, multimedium regional geochemistry in the European Arctic: element concentration,

variation and correlation. Appl. Geochem. 16, 759–780.

Reimann, C., Filzmoser, P., Fabian, K., Hron, K., Birke, M., Demetriades, A., Dinelli,

Ladenberger, A.The GEMAS Project Team, 2012. The concept of compositional

data analysis in practice — total major element concentrations in agricultural

and grazing land soils of Europe. Sci. Total Environ. 426, 196–210

Specific comments:

Abstract, L 44 which depth?

L281-287: This conclusion is rather trivial. The geogenic background is determined by lithology of rocks.

L303 “Lack of information on the mobility of heavy metals..”? Not totally clear what doe sit mean here. There are numerous papers on heavy metals in permafrost lakes (Manasypov, Shirokova), rivers (Krickov, Pokrovsky), soil waters (Raudina) to name just a few, that are devoted to mobility of metals in Arctic and subarctic waters
