# Supplementary material for: Heavy metals in the Arctic: Distribution and enrichment of five metals in Alaskan soils
Source: PLoS One. 2020 Jun 3;15(6):e0233297. doi: 10.1371/journal.pone.0233297 (PMC7269202; doi:10.1371/journal.pone.0233297)
Supplement: Attachment — Submitted filename: Reviewer 1 for PONE-D-19-30690.docx [file pone.0233297.s005.docx]

Reviewer 1.

**Overview:**

Perryman et al. have taken on the challenging task of tabulating and evaluating concentrations of As, Cr, Hg, Ni and Pb in soils from Alaska using data available through the USGS. I believe this is a useful and publishable effort. One common difficulty in such reviews of data is the choice of metals data for comparison from the perspective of (i) health and (ii) typical soils.

I think the health standard values have been reasonably well researched and work relatively well because they are setting a standard, not just summarizing data. Nevertheless, I do think that the health standard of 35 mg/kg for Ni is too low and the health standard for Hg of 6.6 mg/kg is too high. Other data sets show values of ~100 mg/kg for Ni and ~1 mg/kg for Hg (e.g., Kamunda et al., 2016, Intl. J. Env. Research and Pub. Health, doi:10.3390/ijerph3070663). The main product of this exercise is nicely shown in Figure 3 (bar graph). If possible, I prefer that one source be used for the health standards because it now looks like (but surely is not) cherry picking. If any values chosen for a health standard are to be reevaluated, I would recommend doing so for Ni and Hg.

I also recommend that the authors consider using one source for US soil values, preferably from the USGS. I like the data set from Smith et al. (2013, Geochemical and Mineralogical Data for Soils of the Conterminous US, Data Series 801). I have placed a few different sets of values in Table R1. My only real comments here relative to mean values for US soils are (i) best to use one data set, (ii) present choices for Hg and Ni seem low and (iii) present choice for Pb seems high.

Table R1. Selected data for metals in soils.

|  | As  (mg/kg) | Cr (mg/kg) | Hg  (mg/kg) | Ni  (mg/kg) | Pb  (mg/kg) |
| --- | --- | --- | --- | --- | --- |
| US Soil Mean^a^ | 7.2 | 37 | 0.029 | 13 | 30 |
| US Soil Mean A horizon^b^ | **6.6** | **37** | **0.04** | **18.5** | **22.2** |
| US Soil Mean 0-5 cm^b^ | **6.4** | **36** | **0.05** | **17.7** | **25.8** |
| US Soil Mean^c^ | 7.2 | 54 | 0.09 | 19 | 19 |
| Ave. Continental Crust^d^ | 1.7 | 126 | 0.04 | 56 | 14.8 |
|  | | | | | |
| Health Value^a^ | 32 | 100 | 6.6 | 35 | 85 |
| Health Value Canada^e^ | 20 | 250 | 0.8 | 100 | 200 |
|  | | | | | |
| Suspended Matter  Alaskan Rivers^f^ | 12 | 147 | - | 109 | 24 |
| Coastal Marine Sediment  Alaska^g^ | 12 | 62 | 0.040 | 26 | 10 |

^a^Values used in Table S1 from manuscript by Perryman et al.

^b^Values from USGS (Smith et al., 2013, Geochem. and Mineralogical Data for Soils of the Conterminous US, Data Series 801).

^c^Values from USGS for US Soils (Schacklette and Boerngen, 1984; USGS Prof. Paper 1270).

^d^Average crustal rock from Wedepohl (1995, Geochim. Cosmochim. Acta, 59:1217-1232).

^e^From Kamunda et al. (2016, Int J. Env Res and Pub Health, doi:10.3390/ijerph3070663).

^f^Feely et al., 1981, [Distribution and elemental composition of suspended particulate matter in Norton Sound and the northeast Bering Sea Shelf: Implications for Mn and Zn recycling in coastal waters](https://www.pmel.noaa.gov/public/pmel/publications-search/search_abstract.php?fmContributionNum=506). Chapter 20 in *Eastern Bering Sea Shelf: Oceanography and Resources*, D.W. Hood and J.A. Calder (eds.), Vol. 1, USDOC/NOAA/OMPA, 321–337.

^g^Trefry and Neff (2018, Integrate Env. Assessment and Mgt., 15:202-223).

My recommendation to the authors is to consider modifying some of the comparison data for the US soils for comparison with AK soils. These differences do make a significant change to bar graph in Figure 3

In my opinion, the overriding conclusions that (i) “Over 70% of the soil samples had elevated concentrations of As, Cr, Hg and Ni … [lines 273-274) and (ii) “… that elevated concentrations of heavy metals are **pervasive** (too strong with negative connotation) in the Alaskan Artic (*misspelled*) … (line 338) need to be reconsidered and explained before this this paper is published.

In its present form, the paper seems to promote too strongly unwarranted concern about metals in Alaskan soils. For example, only 10.1% of the values for Pb in Alaskan soils were above the average chosen for US soils (Figure 3) because the value of 30 mg/kg used for Pb seems high. In contrast, reporting that 93.5% and 85.8%, respectively, of the concentrations of Hg and Ni in Alaskan soils were above US averages seems driven by choosing low values for US soils.

One additional comment, it seems like there are >200 samples with Hg values that are below detection limits. If so, I recommend that they be included (as say 0.01 mg/kg) in the exercise to show the percent of AK soil samples that are above the US average for soils. The result would change significantly.

******

Yet, the theme of the paper is further complicated (or increased in importance) because previous studies of metals in suspended sediments in AK rivers and in coastal sediments do indeed show enrichment of As, Cr and Ni. Data for river suspended sediments and coastal marine sediments have been shown for many years to be enriched in As, Cr and Ni, but not Hg and Pb (Table R1). I think it would be valuable to bring this story into your discussion. You are reinforcing a point supported by data for river suspended matter and coastal sediments (all derived from upland soils).

Sweeney and Naidu (1989, Mar Poll Bull, 20:140-143) report mean Ni in soils/sediments of 24-31 mg/kg with only 10% extractable. Others have wrestled with higher Ni and Cr values in coastal sediments (a product of soil weathering and transport) in Alaska (e.g., Valette-Silver et al., 1999, Mar Poll Bull 38:702-722; and Trefry and Neff, 2018; Int. Environ. Assess. Mgt. 15:209-223). In other words, available data do suggest that higher concentrations of some metals (e.g., As, Cr and Ni) are found in Alaska. So, while your statement regarding higher metal values in AK soils (and coastal sediments) is on target, these values seem to mostly represent natural conditions and the key point missing (as you address) is the availability of the metals to biota.

**Selected other comments.** Examples of other suggested changes that should be part of a major revision of the manuscript.

Title: Delete present title and consider a title such as “Enrichment of selected (heavy?) metals in soils from the Alaskan Arctic”.

Lines 39-40: Only organic Hg and to a lesser degree organic As have been clearly shown to biomagnify. I recommend taking “biomagnification” out of the Abstract and include brief, similar text as appropriate in the Introduction with mention and references for biomagnification of organic As and Hg (sounds minor, but it looks somewhat unknowledgeable at present).

Lines 42-43: Statement about aiming to assess potential risk from metals in AK soils seems beyond the scope of the paper and would be better left out of Abstract. You are pointing out the enrichment of some metals in AK soils, that’s good enough. In the discussion, you can mention next steps (realizing some of this next layer of information is already available).

Lines 49-54: Final two sentences will likely be revised if some different values are used for comparing US soils with AK soils. I recommend removing the word “pervasive” because of its negative connotation and strong suggestion of anthropogenic perturbations.

The need for “greater understanding” with respect to metal mobilization and exposure has been highlighted for decades with considerable progress in some areas. The point, it has been a comment made for decades. Perhaps, say that the urgency for better understanding of metal remobilization from soils and uptake by organisms has risen to greater importance in light of melting permafrost.

Lines: 74-75. Delete Fe and Mn, sounds like they are “a threat”.

Line 76: Delete Cd, not part of your paper and begs the question “Why not? (I realize that you answer it on line 140; still recommend sticking just to metals that you address).

Lines 82-108: Could use less generalization (e.g., Hg causes neurological damage) and show more current and relevant knowledge pertinent to more specific effects from AK soils.

Line 110: Very few samples in your study are actually from the Alaskan Arctic (i.e., >66.5° N). To avoid a criticism of using Arctic, perhaps add reference that arctic tundra is found from 60-70° N in AK. I would still keep Arctic in the title.

Line 124: Delete Cd and Tl, they are not part of your story. There are certainly many other metals that fit this same pattern; seems awkward to just pick out two other metals.

Figure 1: Show a few lats (e.g., 60, 65, 70) and longs along the side of the map. Consider adding major rivers if it involves just pushing a couple of buttons.

Lines 170f: Consider some revisions to values for health limits and US soils as discussed above.

Line 186-187: Delete Hg concentrations “lower”; common knowledge. If included, then need to address” Why?”

Table 1: Health standards and means for US soils are too important to place in Table S1. I suggest moving those data to Table 1. No more than 2-3 significant figures should be used in Table 1. Could cut IQR if table is getting too busy (you really do not use the IQR).

Line 197. Delete “No clear patterns …” I can see some (e.g., see my Supplement below). However, the impact of mining on As, and Hg is really important right now as AK considers new mining sites such as the Pebble Mine. Be sure to consider emphasizing that point regarding mining. Furthermore, yes, emphasize lack of similarities among other soil types.

Figure 2: Good figure. Ni is listed as Log relative to Ln for other four metals. I prefer Log, easier for people to think about number quickly, your choice.

Lines 214-237. Could take on somewhat new focus. Think about trying to make the seemingly subtle point that enrichment of As, Cr and Ni has also been observed in natural river suspended matter and coastal sediments. However, concentrations of Hg and Pb are low in AK river suspended matter and coastal sediments.

Lines 240ff: Needs a bigger picture look. Imnavait Creek. Why is this comparison being made? Can the observation it be explained?

Line 254: I see too much use of the word “comparable”. All data are comparable. The point is whether means for one set of data are statistically different or not from another set of data.

Line 255: What is detection limit? Link to low Hg in AK? Reminder to consider adding 200? Non-detects to establish number of AK samples with Hg values >US mean.

Figure 3. Important and good. Add lats and longs. Revise bar graph as well as possible. I really do not think the paper should go out with such high percentages for comparison with US averages.

Lines 258-333. Needs a clearer focus and goal. Too much speculation.

Conclusions: Needs some editing and focus on conclusions from your study. Remove gaps from conclusion; if kept, move to Discussion.

************

Supplement:

To gain a better perspective on the soil data for AK, may I suggest that you plot Cr vs Ni (linear scale). Then, create a second plot by adjusting the scales to 0-100 for Cr and 0-60 for Ni. A relatively clear relationship is observed for Cr/Ni on the smaller-scaled graph. The resulting Cr/Ni relationship likely describes background minerals, soils and sediment in AK. Now, go back to the full scale graph. Values for Ni >100 and Cr >150 are most likely due to anthropogenic impact or the presence of different natural source minerals.
