# Supplementary material for: Heavy metals in the Arctic: Distribution and enrichment of five metals in Alaskan soils
Source: PLoS One. 2020 Jun 3;15(6):e0233297. doi: 10.1371/journal.pone.0233297 (PMC7269202; doi:10.1371/journal.pone.0233297)
Supplement: Attachment — Submitted filename: AKSoilMetals_ReviewerResponses.docx [file pone.0233297.s006.docx]

**Reviewer 1:**

The manuscript “Heavy metal in the Arctic” addresses important scientific problem and uses unique set of data which should be presented to the scientific community

However the treatment of the data, comparison with other available studies and discussion of mechanisms and consequences are not at the level suitable for an academic publication

**Response to R1:**

Thank you for your thoughtful and constructive feedback on our manuscript. Your call for a more rigorous comparison of the data we present to other studies has improved our data analysis. We now quantify and report the proportion of samples that are larger than reported averages by a factor of 2, 10, and by one standard deviation (Figure 3), in addition to those that are simply greater or less than the average value. We have revised our definition of “elevated concentrations” to only include those that exceed reported averages for US soils by 1 standard deviation or more.

While we agree that further discussion on the factors controlling the distribution of the five metals we focus on in the manuscript would be interesting, inconsistent metadata reporting in the USGS dataset hinders our ability to explain variation. Rather than discuss the factors that control distribution and mobility of heavy metals in soils experiencing permafrost thaw, our focus for this manuscript is to provide a needed synthesis on the occurrence of heavy metals in Alaskan soils. Through this effort we also highlight gaps in the spatial coverage, depth coverage, and public availability of datasets reporting metal concentrations in soils that preclude a needed pan-Arctic synthesis. We hope our revised manuscript conveys these aims with increased clarity.

Please find our responses to your individual comments below.

**Major issues:**

Since the authors have the data for various depths, they should attempt to estimate the stock of metal sin frozen and seasonally thawed horizons

While the authors agree that this would be a useful and informative inclusion to the manuscript, the data in the database used in our analysis were collected over the span of a few decades (the earliest sample collection year was 1995) which precludes an estimate of the metals stock in frozen and seasonally thawed horizons as the permafrost thaw front and seasonal thaw depth in Alaska has changed significantly over that time (NOAA, [https://arctic.noaa.gov/Report-Card/](https://arctic.noaa.gov/Report-Card/Report-Card-2017/ArtMID/7798/ArticleID/694/Terrestrial-Permafrost)

[Report-Card-2017/ArtMID/7798/ArticleID/694/Terrestrial-Permafrost](https://arctic.noaa.gov/Report-Card/Report-Card-2017/ArtMID/7798/ArticleID/694/Terrestrial-Permafrost)).

The comparison with other studies and with available health standards (L213-237) lacks statistical criteria of exceedance. Sometimes the average (or median) is higher than the health standards but the uncertainty (interquartile, Table 1) are not. The authors should also group the soils by a degree of exceedance (i.e., by a factor of 2, of 10.. etc)

We include an updated Figure 3 that shows the proportion of soils that exceed the average US concentration for each metal by a factor of 2, a factor of 10, and by 1 standard deviation of the reported average, as well as the proportion that are simply larger than the US average from Smith et al. (2013).

The authors often use term “lower or “higher (ex. L 244) when comparing large number of values. The big data base should be compared using very different, and well developed today, statistical criteria. The whole discussion in L 240-255 thus seems to be not supported by rigorous statistical treatment.

We have revised passages of text comparing the data presented in our manuscript to data from other published works acknowledging that the range of values we present are comparable (within the same order of magnitude) to those from previous studies, and if we do indicate that we report values larger than previous reports we indicate the magnitude of difference between the values we report and those to which we are comparing. For example, from lines 253-264:

*“The heavy metal concentrations in the soils in the AGDB2 dataset are within the range of those previously reported in other parts of the Arctic. For example, permafrost-affected soils from the Lena Delta in Siberia have been reported to have median concentrations of As ranging from 1.29 to 11.3 mg kg^-1^, Ni from 5.12 to 30.2 mg kg^-1^, and Pb concentrations of 2.41 to 13.1 mg kg^-1^ [12], which are comparable to the median concentrations of these metals reported in this study (see Table 1). Similarly, the interquartile range (IQR) of concentrations of Cr, Ni and Pb reported in our study (Table 1) are comparable to the range of concentrations reported by Barker et al. [26] from a soil core from the Imnavait Creek watershed in Alaska, which were 10.7 - 142.7 mg kg^-1^, 13.9 - 23.7 mg kg^-1^ and 4.8 - 13.1 mg kg^-1^, respectively. Halbach et al. [44] measured a suite of metal concentrations in Svalbard, and they report median As, Cr, and Ni concentrations approximately 2 to 4 times lower than the median concentrations reported in this study and Hg and Pb concentrations comparable to the median values in our database.”*

Another example is L272-274. Such comparison of average values is not acceptable. The natural range of metal concentrations is at least 1 order of magnitude. Does this above mean > 10 times higher? If not, it is not necessarily ‘higher’, and serious statistical treatment is needed to judge the difference.

We have revised our definition of “elevated concentrations” to be those that are greater than the average value for A Horizon soils in the USA reported in Smith et al. (2013) by one standard deviation (using the standard deviation for each average reported in Smith et al., 2013). Figure 3 indicates that for all 5 metals discussed in our manuscript, one standard deviation above the reported US average is approximately as large of an exceedance as 10x the national average. We chose to use an exceedance criteria of 1 standard deviation to ground our definition in the variation reported from soils from other regions.

The PCA (and HCA) treatment of metal concentrations together with other soil properties may be useful to reveal controlling factors and correlations between elements (and other soil parameters)

We attempted this analysis in the original data preparation for this manuscript. The lack of consistent metadata beyond sample source and depth (e.g., soil pH, soil carbon, texture, composition) prevented a robust and informative analysis of the controlling factors of metal concentrations.

L288-333: This text represents some review/perspectives of research, not directly linked to the obtained results. Part of this text can be in the Introduction, but the Discussion should be focused on results of this study and explanation of the pattern of metal distribution in soils with respect to landscape parameters, anthropogenic factors, rock lithology, permafrost distribution, soil type etc. Examples for the Scandinavian Arctic soils:

We have condensed and moved this section of the text into the second paragraph of the introduction (L71-85). We now use our discussion to highlight gaps in available data on heavy metals in Arctic and sub-Arctic soils we found through our efforts to synthesize these data. Outlining these gaps serves as a roadmap for where future work should focus in order to better constrain the pool and fate of heavy metals in soils and ecosystems in a rapidly warming Arctic.

Reimann, C., de Caritat, P., 2005. Distinguishing between natural and anthropogenic

sources for elements in the environment: regional geochemical surveys versus

enrichment factors. Sci. Total Environ. 337, 91–107

Reimann, C., Banks, D., de Caritat, P., 2000a. Impacts of airborne contamination on

regional soil and water quality: the Kola Peninsula, Russia. Environ. Sci. Technol.

34, 2727–2732.

Reimann, C., Banks, D., Kashulina, G., 2000b. Processes influencing the chemical

composition of the O-horizon of podzols along a 500 km north-south profile

from the coast of the Barents Sea to the Arctic Circle. Geoderma 95, 113–139.

Reimann, C., Kashulina, G., de Caritat, P., Niskavaara, H., 2001. Multi-element, multimedium regional geochemistry in the European Arctic: element concentration,

variation and correlation. Appl. Geochem. 16, 759–780.

Reimann, C., Filzmoser, P., Fabian, K., Hron, K., Birke, M., Demetriades, A., Dinelli,

Ladenberger, A.The GEMAS Project Team, 2012. The concept of compositional

data analysis in practice — total major element concentrations in agricultural

and grazing land soils of Europe. Sci. Total Environ. 426, 196–210

Specific comments:

Abstract, L 44 which depth?

L281-287: This conclusion is rather trivial. The geogenic background is determined by lithology of rocks.

We acknowledge that heavy metals in soils come from many sources (lithologic, anthropogenic, etc.), and that geogenic background concentrations of heavy metals in Alaska contribute to higher concentrations in soils. While the source of these metals is not the main point of our work we feel that we need to acknowledge that mine-impacted sites are not driving the results of our analysis (given their high concentration of all metals, see Fig S1).

L303 “Lack of information on the mobility of heavy metals..”? Not totally clear what doe sit mean here. There are numerous papers on heavy metals in permafrost lakes (Manasypov, Shirokova), rivers (Krickov, Pokrovsky), soil waters (Raudina) to name just a few, that are devoted to mobility of metals in Arctic and subarctic waters

We have omitted this sentence in the revised manuscript.

**Reviewer 2:**

The manuscript presents relevant new information and has high quality. However, the manuscript presents few points that need to be revised. The detailed comments are listed below:

**Response to R2:**

Thank you for providing a review of our manuscript. We have made the revisions to the text you suggest, the details of which you can find in our responses to line items below. Furthermore, we provided greater context in our discussion section of the implications of high concentrations of the 5 metals we discuss throughout the study region in the discussion, particularly how they relate to previous work from Alaska that suggests heavy metals in freshwater resources and sediments have a terrestrial source.

Keywords

1. Delete all keywords already in title.

Done.

Introduction

1. Line 114 à Delete “to”.
Done.

Materials and Methods

1. Line 128: Delete “Alaskan Geochemical Database Version 2.0”.

Done.

Results

1. Table 1 presentation should be improved.

Mean concentration values and US average soil concentrations were added to Table 1 and the units of mg/kg have been changed to mg kg^-1^.

Discussion

1. Line 243 and all manuscript: mg/kg à mg kg-1.

Done.

2. Second paragraph: What implications can these results have on the environment?

Transfer to surface and subsurface waters?

We added the following text within this paragraph in L324-336 to address the transfer of metals from hydrologic changes as per your suggestion and the suggestion of Reviewer 4:

*“Arctic communities are particularly susceptible to threats to water security from climate change [28], and potential liberation of metals through permafrost thaw may additionally threaten water quality in the Arctic [7,27]. Our study shows that high concentrations of heavy metals are distributed in soils across Alaska, suggesting permafrost thaw and associated changes in the environment may present an unaccounted-for pathway of heavy metal exposure. This conclusion is supported by previous observations in Alaska of suspended river and coastal sediments enriched in heavy metals [54,55], increased elemental and sediment loading into freshwater ecosystems as permafrost thaws [56,57], and of a seasonal signature of stream water metal concentrations that coincides with the depth of seasonal thaw [27]. Given recent observations of widespread and rapid permafrost thaw due to ongoing warming [58] and increases in wildfires that accelerate thermokarst expansion [59], more attention to the threat of heavy metal liberation from thawing permafrost is needed.”*

Line 310: It does not reduce SOM's ability to retain metals, it only reduces its soil contents. Rewrite.

The revised manuscript presents information on the mobility of metals previously in the discussion in a condensed form in the Introduction L71-85. On this point, we now state:

*“Additionally, increases in soil organic matter (SOM) degradation during permafrost thaw can impact metal mobility as metals bound to SOM are released [13].”*

**Reviewer 3:**

Reviewer #3: PLOS ONE

Manuscript Number: PONE-D-19-30690

Title: Heavy metal in the Arctic: Characterizing the distribution of five metals in soil in the Alaskan Arctic

The article is clearly presented and straightforward. While the authors did not synthesize any of their own data, they do present a large publically available dataset in a concise way that has not been done before for Alaska. Particularly, the key finding that there is a bias in sampling towards the upper 10 cm of soils and that may be biasing values to be preferentially lower for some metals versus others that tend to accumulate more in the deeper subsurface is an important finding.

Overall, comments are relatively minor. There are two major edits that need to be addressed: (1) the authors need to be more clear when comparing Alaska values to ‘US averages’ and ‘health standards’, it is confusing and somewhat misleading as written (explained further below in the line items) and (2) the authors need to clarify background values for the different classification ‘bin’ types – there should be published values on background concentrations for Alaska soils (also would be important to compare to crustal abundances for the metals studied).

**Response to R3:**

Thank you very much for your thoughtful feedback, it has helped us produce a revised manuscript with increased clarity and relevance. We have further clarified and strengthened our comparison to US soil averages in the revised manuscript, and these changes are detailed in the responses to your individual comment below. We elected to omit a comparison to health standards in the revised manuscript for clarity, and further rationale for that decision is also explained below in response to your specific comments. The revised manuscript provides greater clarity in describing our methodology for grouping land cover types. In the revised manuscript we also show that the values we present are comparable to studies conducted across Arctic sites (including those that include some sampling of deeper, soils and those conducted in remote areas representing “background” levels), indicating that sites affected by contamination are not driving our results. Please see more detailed responses to your individual comments below.

Line items:

Title: Is there a reason the first ‘metal’ mentioned is not plural – i.e. ‘Heavy metals in the Arctic’ – sounds odd as it is since you are studying more than one metal.

We have revised the title to “*Heavy metals in the Arctic: Distribution and enrichment of five metals in Alaskan soils*” to better reflect the contents of the paper and avoid word play.

Lines 62-64: I would clarify that there are also heavy metals present in the Arctic naturally in the soils at low concentrations in addition to anthropogenic sources.

We have revised the text to further clarify the different origins of heavy metals in Arctic soils, highlighting that natural sources can result in detectable levels of these metals in soils unrelated to anthropogenic activity.

Introduction, L66-67:

*“Heavy metals are present in Arctic soils due to weathering [8], atmospheric deposition [9], and anthropogenic activities including mining and/or smelting [10,11].”*

In L295-298 of the Discussion, we also address this point:

*“Aside from anthropogenic contamination, heavy metals in soils can be derived from lithogenic pools that become distributed across surface environments through natural biogeochemical cycles and near-surface processes (i.e. erosion, weathering, pedogenesis) [51].”*

Line 83: consider changing ‘peoples’ to ‘communities’

Lines 86 and 90: consider changing ‘country’ to subsistence-derived’

L86-97: Country foods is a common term used in the literature (see DOIs *10.2993/0278-0771-38.3.333, 10.1016/j.jand.2018.02.004, 10.1007/s10393-018-1348-z, 10.1016/B978-0-12-817945-1.00024-1*). Therefore we will keep the terminology as is. Regarding the suggested change from *peoples* to *communities*, we will also keep the wording as is. Country foods are most prevalent among Inuit, which is reflected by the word peoples. Non-natives are less prone to foraging even though they might be part of the community. Our wording aims to make this distinction.

Line 95: is there a verb missing between ‘and’ and ‘substantial’?

We have changed the phrasing for clarity. L98-100:

*“We focus on five heavy metals that have both documented occurrences in Arctic food and water resources and have substantial human health effects: arsenic (As), chromium (Cr), mercury (Hg), nickel (Ni), and lead (Pb).”*

Lines 150-153: Can you go more in depth on the classification systems that you re-coded? For the re-coded ‘tundra’ category does the included ‘shrub-covered’ and ‘grassland/grazing’ mean there were underlain with permafrost? Go more in-depth about the original USGS land-cover categories and what they meant and why you chose to include those groups together. I would assume the ‘tundra’ re-coding includes only places with permafrost? As it is written, it is unclear what those categories actually mean.

We have revised the methods to better reflect the original and modified data labels which were used for land cover type. All of the “sample source type” as they were coded in the USGS database were self reported over the collection period (c. 1995-2009) and varied in their content. Some referred to drill/boring type, others referred to land cover type, and even more were left blank. During data cleaning the nearly 40 self reported sample source labels were coarsened to the land cover type labels used in the manuscript. The grouping was done by combining closely related original labels (ex. upland forest and forest) into broader land cover categories, using recent land cover inventories for reference (Walker et al., 2005; NLCD, 2011). Additionally, the inconsistency of the original self reported labels and the timeframe across which they were created makes drawing conclusions about their status as permafrost tenuous at best, and therefore we refrained from classifying land cover based on permafrost zone. See L154-162 in the revised manuscript for updated text in the Methods section regarding the land cover types.

Line 165: What do you mean by ‘there are no conclusive U.S. standards’… for what? Drinking water? Soils? Fish? The US has drinking water standards, soil standards, etc. What do you mean by that statement?

We address this in our revised discussion, L274-284. We elected to omit comparison to health guidelines in the revised manuscript given the large (one to two orders of magnitude) difference in health standards globally for the metals we report (see Kamunda et al., 2016; Table 4). As such, the result of comparing soil concentrations to health standards for any of the metals is highly influenced by the variation in these standards globally and the (largely subjective, given their variability) selection of standards by the research team.

Lines 171-172: Please discuss the criteria for how ATSDR determined the average concentrations of these metals in soils. It will help with your later comparisons for Alaska. Was Alaska included in the ATSDR calculations?

We are no longer using ATSDR standards for comparison. In the revised manuscript we use average concentrations for A Horizon soils from the contiguous United States reported by Smith et al. (2013) (USGS Data Series 801; <https://pubs.usgs.gov/ds/801/pdf/ds801.pdf>) that do not include Alaska.

Figure 2: resolution looks blurry – it looks different than the other images in terms of resolution

This was a formatting and post-processing problem which we have resolved in the revised manuscript.

Line 223: Which human health standard are you referring to? This whole paragraph refers to human health standards and US averages – list those standards and averages for referring to

We no longer compare the data from the AGDB2 to health standards and instead focus on comparison to US soil averages from Smith et al. (2013) in the results section beginning on L225.

Figure 3: Alaska is known to have high arsenic values naturally in their soils (in addition to mining activities) – I think it’s important to note the background values for all these metals. Arsenic in Alaska, for example, is always going to have higher than US average soils because there is an abundance of arsenic in soils up here – not necessarily due to ‘contamination’ – that is known. There needs to be a discussion of background values and crustal abundances for all the metals examined here. Also, please include what ‘health limit’ you are referring to on the figure somewhere – either in the caption or on the plot. The value and also what type of limit – drinking water, etc. It is very unclear, as is.

We have revised the manuscript throughout to focus less on “contamination” and highlight that the metals in soils in Alaska come from a myriad of natural sources in addition to anthropogenic sources. Furthermore, we discuss in L285-300 how the sampling bias towards surface (upper 10 cm) soils impeded an assessment of background levels in these soils, given the wide distribution of sites with known contamination in Alaska (Fig S2). During our revisions, we performed a literature search for background (pristine, lithogenic, crustal, etc.) metal concentration values for Alaska to provide greater context for our findings, but due to the heterogeneous bedrock geology in this region we did not find values representative of the whole study region.

Discussion, 1st paragraph: Please note somewhere within this text that you are comparing average values for the entire state of Alaska (including legacy and active mining sites and likely some military sites) with concentrations from pristine sites (citations 12, 13, 45, 46) – the values should be different so that is not a new finding. The paragraph is a bit misleading as written – what would be more appropriate is to compare areas in Alaska with no known anthropogenic sources (Hg will be difficult to do this with) to the citations 12, 13, 45, and 46 and vice versa – compare legacy contamination with the ‘high’ values found in this report. Or only look at the ‘arctic’ values where there is little contamination.

The revised version of this paragraph (L253-269) that incorporates feedback from Reviewer 1 regarding the rigor of our comparisons to previous work shows that the median and IQR for the concentrations of As, Cr, Hg, Ni, and Pb are comparable (within the same order of magnitude or closer) to those reported from other sites. Furthermore, Fig S2 shows that the proportion of samples we identify as having “elevated concentrations” (> US average + 1 standard deviation) is not driven by the inclusion of the small number (< 20) of mine-impacted sites in the data set.

Methods section: you mentioned that you separated published values based on analytical method – any information on that in the document? Are some analyses different than others? There should be some mention of that in the results, etc.

The AGDB2 “Best Value Soil” dataset we used in our analysis was pre-screened by USGS chemists for quality control. Their “best value” ranking system selected the method providing the most accurate value for each sample that had been analyzed using more than one analytical instrument as follows:

“Different analytical determinations of the same element for any given sample do not have equal value to a user for different purposes. Some analytical methods result in values that are more quantitative, precise, or accurate than others. When developing a ranking of methods best for any particular use it is necessary to take into account factors which vary between analytical methods. These include weight of sample analyzed, method of decomposition of the sample during the preparation for analysis, sensitivity and accuracy of the instrument used in each method, upper and lower limits of detection for a given element by a given method, the age of the method and stage of its development when a specific analysis was performed, and the exact analytical laboratory and equipment used. Creation of the AGDB2 “best value” subset followed a rubric or decision tree that considered all these factors in ranking the analytical methods in the AGDB2 to determine methods most useful to mineral resource evaluation.” (<https://pubs.usgs.gov/ds/759/contents/DS759_pamphlet.pdf>)

We added information about the “Best Value” selection in the Methods section L144-147.

The majority of values for chromium, nickel, and lead in the AGDB2 data used in our analysis were collected using inductively coupled plasma-atomic emission spectrometry and inductively coupled plasma-mass spectrometry. Arsenic was primarily collected using hydride generation-atomic absorption spectrometry and inductively coupled plasma-mass spectrometry. All values we report for mercury were collected using cold vapor-atomic absorption spectrophotometry. Values in the “Best Value” dataset we used in our analysis employ digestions during sample preparation that yield approximate “total” analyses of the elements of interest.

Analytical method does not co-vary with land cover type or depth; and therefore differences in concentrations reported between the analytical methods do not skew our reporting of the patterns in metal concentrations across the landscape or through the soil profile.

Section ‘Comparison to health standards and US averages’: Does the US average include Alaska and Hawaii samples or not?

The USGS A horizon soil averages are for the contiguous United States.

Line 262: except Hg? You mentioned earlier that Hg was higher in the 0-10 cm bin.

Thank you for this. This was a typo in the Results of the previously submitted manuscript that made these sentences inconsistent. We have corrected it in the revised manuscript. Hg is highest in the > 30 cm depth bin.

Lines 268-271: Remove or reword the statement about permafrost acting as a barrier to metal migration – this statement as written assumes the metal concentrations found in the two studies mentioned (Antcibor and Barker) migrated down into permafrost and that is why the concentrations are higher. Some of these soils are very old and are a result of glacial deposits or wind-blown deposition processes over time and not as a result of metals leaching downward from the surface.

We revised this sentence to state (L314-318):

*“In two studies that did investigate deeper soil horizons, metals were found to be higher both just above the permafrost table [13] and within permafrost [27], a reflection that the prevalence of heavy metals in permafrost soil profiles may be complicated by site lithology and hydrology, mobility of the heavy metal in question, and previous thaw history.”*

Line 291: Include citation for arsenic mobility, same for the next line on chromium

We omitted these sentences in the revision of the manuscript because we chose to condense our discussion of what influences the ability of metals to be liberated from soils. Per reviewer 1, this was also moved into the second paragraph of the Introduction of the manuscript.

Reviewer 4:

**Overview:**

Perryman et al. have taken on the challenging task of tabulating and evaluating concentrations of As, Cr, Hg, Ni and Pb in soils from Alaska using data available through the USGS. I believe this is a useful and publishable effort. **One common difficulty in such reviews of data is the choice of metals data for comparison from the perspective of (i) health and (ii) typical soils.**

I think the health standard values have been reasonably well researched and work relatively well because they are setting a standard, not just summarizing data. Nevertheless, I do think that the health standard of 35 mg/kg for Ni is too low and the health standard for Hg of 6.6 mg/kg is too high. Other data sets show values of ~100 mg/kg for Ni and ~1 mg/kg for Hg (e.g., Kamunda et al., 2016, Intl. J. Env. Research and Pub. Health, doi:10.3390/ijerph3070663). The main product of this exercise is nicely shown in Figure 3 (bar graph). If possible, I prefer that one source be used for the health standards because it now looks like (but surely is not) cherry picking. If any values chosen for a health standard are to be reevaluated, I would recommend doing so for Ni and Hg.

I also recommend that the authors consider using one source for US soil values, preferably from the USGS. I like the data set from Smith et al. (2013, Geochemical and Mineralogical Data for Soils of the Conterminous US, Data Series 801). I have placed a few different sets of values in Table R1. My only real comments here relative to mean values for US soils are (i) best to use one data set, (ii) present choices for Hg and Ni seem low and (iii) present choice for Pb seems high.

**Response to R4:**

Thank you for your thoughtful and thorough review of our manuscript. Your suggestions have helped us produce a much improved and more nuanced paper.

We have revised our comparison of the values in the AGDB2 dataset to published averages for US soils to use the Smith et al. (2013) values for US soil mean A horizon. Thank you for your suggestion. We also present this comparison in the revised manuscript in an updated figure that shows the proportion of soils that exceed the average US concentration for each metal by a factor of 2, a factor of 10, and by 1 standard deviation of the reported mean, as well as the proportion that are simply larger than the US average, the latter of which was in our original analysis. This amendment was based on your comments and the suggestions of Reviewer 1. We now define “elevated concentrations” as those that exceed the average reported in Smith et al. (2013) by more than 1 standard deviation. Under this definition 3.7% to 18.7% of the samples had “elevated concentrations” of the selected metals. We have elected to omit a comparison to health standards due to the wide variability in these standards globally; further discussion of this decision can be found in L270-284.

We have also incorporated your suggestion to bring previous work on metals in suspended and coastal sediments into our discussion for broader context of our results, and this revision has improved helped strengthen the central ideas in our manuscript. We appreciate the care you took in providing thorough feedback. Our responses detailing the revisions to the manuscript we made in light of your specific comments can be found below.

Table R1. Selected data for metals in soils.

|  | As  (mg/kg) | Cr (mg/kg) | Hg  (mg/kg) | Ni  (mg/kg) | Pb  (mg/kg) |
| --- | --- | --- | --- | --- | --- |
| US Soil Mean^a^ | 7.2 | 37 | 0.029 | 13 | 30 |
| US Soil Mean A horizon^b^ | **6.6** | **37** | **0.04** | **18.5** | **22.2** |
| US Soil Mean 0-5 cm^b^ | **6.4** | **36** | **0.05** | **17.7** | **25.8** |
| US Soil Mean^c^ | 7.2 | 54 | 0.09 | 19 | 19 |
| Ave. Continental Crust^d^ | 1.7 | 126 | 0.04 | 56 | 14.8 |
|  | | | | | |
| Health Value^a^ | 32 | 100 | 6.6 | 35 | 85 |
| Health Value Canada^e^ | 20 | 250 | 0.8 | 100 | 200 |
|  | | | | | |
| Suspended Matter  Alaskan Rivers^f^ | 12 | 147 | - | 109 | 24 |
| Coastal Marine Sediment  Alaska^g^ | 12 | 62 | 0.040 | 26 | 10 |

^a^Values used in Table S1 from manuscript by Perryman et al.

^b^Values from USGS (Smith et al., 2013, Geochem. and Mineralogical Data for Soils of the Conterminous US, Data Series 801).

^c^Values from USGS for US Soils (Schacklette and Boerngen, 1984; USGS Prof. Paper 1270).

^d^Average crustal rock from Wedepohl (1995, Geochim. Cosmochim. Acta, 59:1217-1232).

^e^From Kamunda et al. (2016, Int J. Env Res and Pub Health, doi:10.3390/ijerph3070663).

^f^Feely et al., 1981, [Distribution and elemental composition of suspended particulate matter in Norton Sound and the northeast Bering Sea Shelf: Implications for Mn and Zn recycling in coastal waters](https://www.pmel.noaa.gov/public/pmel/publications-search/search_abstract.php?fmContributionNum=506). Chapter 20 in *Eastern Bering Sea Shelf: Oceanography and Resources*, D.W. Hood and J.A. Calder (eds.), Vol. 1, USDOC/NOAA/OMPA, 321–337.

^g^Trefry and Neff (2018, Integrate Env. Assessment and Mgt., 15:202-223).

My recommendation to the authors is to consider modifying some of the comparison data for the US soils for comparison with AK soils. These differences do make a significant change to bar graph in Figure 3

In my opinion, the overriding conclusions that (i) “Over 70% of the soil samples had elevated concentrations of As, Cr, Hg and Ni … [lines 273-274) and (ii) “… that elevated concentrations of heavy metals are **pervasive** (too strong with negative connotation) in the Alaskan Artic (line 338) need to be reconsidered and explained before this this paper is published.

In its present form, the paper seems to promote too strongly unwarranted concern about metals in Alaskan soils. For example, only 10.1% of the values for Pb in Alaskan soils were above the average chosen for US soils (Figure 3) because the value of 30 mg/kg used for Pb seems high. In contrast, reporting that 93.5% and 85.8%, respectively, of the concentrations of Hg and Ni in Alaskan soils were above US averages seems driven by choosing low values for US soils.

Our revised analysis is consistent with this initial conclusion, described above in (i). Using the Smith et al. (2013) A horizon values, over 70% of the samples are above mean values for US soils for arsenic (76.8%), chromium (70.1%), mercury (71%), and nickel (75.5%). Furthermore, over 25% of samples have concentrations of over double the mean value for US soils for arsenic (40.3%), chromium (28.4%), mercury (26.1%), and nickel (32.2%). In the revised manuscript, we elected to define “elevated” concentrations as those that exceed the average value reported by Smith et al. (2013) by one standard deviation or more (using the standard deviations reported by Smith et al.). Under this definition 3.7% to 18.7% of the samples had “elevated concentrations” of the selected metals. Because of changes to the analysis, we have now moderated the tone of the manuscript, particularly in reference to your concern in (ii), when we discuss the number of samples above averages.

One additional comment, it seems like there are >200 samples with Hg values that are below detection limits. If so, I recommend that they be included (as say 0.01 mg/kg) in the exercise to show the percent of AK soil samples that are above the US average for soils. The result would change significantly.

The difference between the number of observations we report for mercury and the other metals is not a result of our data cleaning. Prior to removing values < 0, mercury had missing (NA) values for 305 out of 1151 samples in the dataset (at this point samples without sample source or depth information had already been removed). After removing values < 0, the number of NAs in the column for mercury only increased from 305 to 350. As a comparison, arsenic, chromium, and nickel each only had ~70 missing (NA) values prior to the removal of values < 0.

Currently, 71% (568/800) of the samples with Hg values that passed our data QC are greater than the Smith et al. (2013) reported average of 0.04 mg kg^-1^. Adding in the 45 Hg data points we removed from the dataset due to their negative values would only change this result by 4% (568/845). The impact on our new working definition of “elevated concentration” of Hg (greater than 0.04 + 0.17 mg kg-1; Smith et al., 2013) is even smaller - adding in the 45 removed negative values only decreases the proportion of the samples with elevated Hg from 9.2% (73/800) to 8.6% (73/845).

******

Yet, the theme of the paper is further complicated (or increased in importance) because previous studies of metals in suspended sediments in AK rivers and in coastal sediments do indeed show enrichment of As, Cr and Ni. Data for river suspended sediments and coastal marine sediments have been shown for many years to be enriched in As, Cr and Ni, but not Hg and Pb (Table R1). I think it would be valuable to bring this story into your discussion. You are reinforcing a point supported by data for river suspended matter and coastal sediments (all derived from upland soils).

Sweeney and Naidu (1989, Mar Poll Bull, 20:140-143) report mean Ni in soils/sediments of 24-31 mg/kg with only 10% extractable. Others have wrestled with higher Ni and Cr values in coastal sediments (a product of soil weathering and transport) in Alaska (e.g., Valette-Silver et al., 1999, Mar Poll Bull 38:702-722; and Trefry and Neff, 2018; Int. Environ. Assess. Mgt. 15:209-223). In other words, available data do suggest that higher concentrations of some metals (e.g., As, Cr and Ni) are found in Alaska. So, while your statement regarding higher metal values in AK soils (and coastal sediments) is on target, these values seem to mostly represent natural conditions and the key point missing [Office2] (as you address) is the availability of the metals to biota.

Lines 214-237. Could take on somewhat new focus. Think about trying to make the seemingly subtle point that enrichment of As, Cr and Ni has also been observed in natural river suspended matter and coastal sediments. However, concentrations of Hg and Pb are low in AK river suspended matter and coastal sediments.

We have included this important context into our discussion in L324-333:

*“Arctic communities are particularly susceptible to threats to water security from climate change [28], and potential liberation of metals through permafrost thaw may additionally threaten water quality in the Arctic [7,27]. Our study shows that high concentrations of heavy metals are distributed in soils across Alaska, suggesting permafrost thaw and associated changes in the environment may present an unaccounted-for pathway of heavy metal exposure. This conclusion is supported by previous observations in Alaska of suspended river and coastal sediments enriched in heavy metals [54,55], increased elemental and sediment loading into freshwater ecosystems as permafrost thaws [56,57], and of a seasonal signature of stream water metal concentrations that coincides with the depth of seasonal thaw [27].”*

**Selected other comments.** Examples of other suggested changes that should be part of a major revision of the manuscript.

Title: Delete present title and consider a title such as “Enrichment of selected (heavy?) metals in soils from the Alaskan Arctic”.

The revised manuscript is titled: *“Heavy metals in the Arctic: Distribution and enrichment of five metals in Alaskan soils”*

Lines 39-40: Only organic Hg and to a lesser degree organic As have been clearly shown to biomagnify. I recommend taking “biomagnification” out of the Abstract and include brief, similar text as appropriate in the Introduction with mention and references for biomagnification of organic As and Hg (sounds minor, but it looks somewhat unknowledgeable at present).

Lines 42-43: Statement about aiming to assess potential risk from metals in AK soils seems beyond the scope of the paper and would be better left out of Abstract. You are pointing out the enrichment of some metals in AK soils, that’s good enough. In the discussion, you can mention next steps (realizing some of this next layer of information is already available).

We have revised the opening on the Abstract as follows:

*“Metal contamination of food and water resources is a known public health issue in Arctic and sub-Arctic communities due to the proximity of many communities to mining and drilling sites. In addition, permafrost thaw may liberate heavy metals sequestered in previously frozen soils, potentially exacerbating contamination of food and water resources by increasing the concentration of metals in freshwater, plants, and wildlife. Here we assess the enrichment of selected heavy metals in Alaskan soils by synthesizing publicly available data of soil metal concentrations.”*

Lines 49-54: Final two sentences will likely be revised if some different values are used for comparing US soils with AK soils. I recommend removing the word “pervasive” because of its negative connotation and strong suggestion of anthropogenic perturbations.

The need for “greater understanding” with respect to metal mobilization and exposure has been highlighted for decades with considerable progress in some areas. The point, it has been a comment made for decades. Perhaps, say that the urgency for better understanding of metal remobilization from soils and uptake by organisms has risen to greater importance in light of melting permafrost.

We have revised the end of our abstract in line with our updated results as follows:

*“Arsenic, chromium, mercury, nickel and lead concentrations exceeded average values for US soils by one standard deviation or more in 3.7% to 18.7% of the samples in this dataset. We found substantial clustering of sample sites in the southwestern portion of Alaska in discontinuous and sporadic permafrost, while the more populous Interior and continuous permafrost zone in Northern Alaska are severely understudied. Our analysis highlights critical gaps that impede understanding of how heavy metals in thawing permafrost soils may become mobilized and increase exposure risk for Arctic communities.”*

Lines: 74-75. Delete Fe and Mn, sounds like they are “a threat”.

We have revised these sentences as follows (L75-80):

*“Elevated Hg concentrations have been found downstream of thaw slumps [7], illustrating the potential for permafrost thaw to pose added danger to human health as this Hg may infiltrate water and food resources. Other metals may be similarly liberated by permafrost thaw. High concentrations of iron, manganese, nickel, and zinc have been found in permafrost-affected soils in northern Siberia [13], and elevated concentrations of iron, aluminum, chromium, and lead have been observed in areas that experienced rapid thaw [6].”*

Line 76: Delete Cd, not part of your paper and begs the question “Why not? (I realize that you answer it on line 140; still recommend sticking just to metals that you address).

Done.

Lines 82-108: Could use less generalization (e.g., Hg causes neurological damage) and show more current and relevant knowledge pertinent to more specific effects from AK soils.

We have condensed this section of the manuscript, L100-105.

Line 110: Very few samples in your study are actually from the Alaskan Arctic (i.e., >66.5° N). To avoid a criticism of using Arctic, perhaps add reference that arctic tundra is found from 60-70° N in AK. I would still keep Arctic in the title.

We have revised this statement to say:

*“As such, in this study we synthesize available data on the concentration of As, Cr, Hg, Ni, and Pb in soils in Alaska between 60-70°N, where arctic tundra is prevalent, to assess the occurrence of elevated metal concentrations and the risk of heavy metal exposure in permafrost-affected soils in this region.”* (L118-121)

Line 124: Delete Cd and Tl, they are not part of your story. There are certainly many other metals that fit this same pattern; seems awkward to just pick out two other metals.

We have revised the methods to exclude discussion of our search for data on soil Cd and Tl that did not yield results reported in this manuscript.

Figure 1: Show a few lats (e.g., 60, 65, 70) and longs along the side of the map. Consider adding major rivers if it involves just pushing a couple of buttons.

We have added a map grid which has improved the overall readability and quality of the maps. Thank you very much for this suggestion. Unfortunately, adding major rivers was not feasible. We had experimented with this at an earlier stage. Because freshwater is so abundant in Alaska, adding it to the map decreased readability because it obscured the data points we aimed to show on the map figures.

Lines 170f: Consider some revisions to values for health limits and US soils as discussed above.

Revisions/omissions of these values in the revised manuscript discussed above.

Line 186-187: Delete Hg concentrations “lower”; common knowledge. If included, then need to address” Why?”

We have omitted this sentence from the revised manuscript.

Table 1: Health standards and means for US soils are too important to place in Table S1. I suggest moving those data to Table 1. No more than 2-3 significant figures should be used in Table 1. Could cut IQR if table is getting too busy (you really do not use the IQR).

We have edited the table to include the soil averages used in our analysis. We use IQR in our revised discussion to compare to previous work and have retained that information in Table 1.

Line 197. Delete “No clear patterns …” I can see some (e.g., see my Supplement below). However, the impact of mining on As, and Hg is really important right now as AK considers new mining sites such as the Pebble Mine. Be sure to consider emphasizing that point regarding mining. Furthermore, yes, emphasize lack of similarities among other soil types.

We have revised this section as follows (L206-216):

*“Metal concentrations varied between sampling depth intervals of 0-10 cm, 10-30 cm, and below 30 cm for all metals except As (Fig 2). While Ni concentrations are highest above 10 cm (χ2[2] = 63.38, p < 0.001; Kruskal-Wallis multiple comparisons of means and Fisher’s Least Significant Difference tests for all comparisons reported here), all other metals reported in this study have higher concentrations at depth. Concentrations are highest in the 10-30 cm depth interval for Cr (χ2[2] = 43.14, p < 0.001) and Pb (χ2[2] = 11.39, p = 0.003) and below 30 cm for Hg (χ2[2] = 6.68, p = 0.035). Metal concentrations also varied between land cover types (S1 Fig). Wetland sites had the lowest concentration of both As and Pb (χ2[4] = 99.76, p < 0.001; χ2[4] = 11.91, p < 0.001). Forested sites had higher concentrations of Cr and Ni and lower concentrations of Hg than other vegetated land cover types. Mine-impacted soils have higher concentrations of all metals except Ni, for which mine-impacted sites and forested sites had comparable concentrations (p < 0.001 for all).”*

Figure 2: Good figure. Ni is listed as Log relative to Ln for other four metals. I prefer Log, easier for people to think about number quickly, your choice.

We edited the figure to correct this typo. Furthermore, we have opted to include the boxplots showing the differences between sample depths in the main text as Figure 2 and have moved this figure comparing metal concentration between land cover types into the supplement (Figure S1).

Lines 240ff: Needs a bigger picture look. Imnavait Creek. Why is this comparison being made? Can the observation it be explained?

We wanted to include at least one study of soil metals conducted in Alaska, as much of the previous work has been conducted in Scandinavia, Siberia, and the High Arctic.

Line 254: I see too much use of the word “comparable”. All data are comparable. The point is whether means for one set of data are statistically different or not from another set of data.

We have revised the 1st paragraph of the discussion to include more precise language when comparing the values in the AGDB2 dataset to other previous work. These revisions are described in more detail in response to Reviewer 1.

Line 255: What is detection limit? Link to low Hg in AK? Reminder to consider adding 200? Non-detects to establish number of AK samples with Hg values >US mean.

USGS detection limit for Hg if 0.01 mg/kg. We address the difference between the number of observations for Hg and the other metals above (i.e. Hg is not lower due to our data cleaning removing values representing those below detection).

Figure 3. Important and good. Add lats and longs. Revise bar graph as well as possible. I really do not think the paper should go out with such high percentages for comparison with US averages.

We have revised this figure to include a bar graph showing the percent of samples greater than USGS averages reported in Smith et al. (2013), as well as those greater than those averages by a factor of 2, a factor of 10, and by one standard deviation. The revised maps now indicate the location of samples that are larger than the USGS reported averages by 1 standard deviation or more. We have also added latitude and longitude gridlines.

Lines 258-333. Needs a clearer focus and goal. Too much speculation.

We have significantly revised the discussion to focus on the data gaps we identified through our attempt at a data synthesis and placing our results in context of previous studies quantifying metals in soils, sediments, and freshwater, as per your suggestions. Previous commentary on the mobility of metals has been condensed and moved to the Introduction.

Conclusions: Needs some editing and focus on conclusions from your study. Remove gaps from conclusion; if kept, move to Discussion.

We have included more discussion of the data gaps we identify in the Discussion section (L297-315).

************

Supplement: and also

To gain a better perspective on the soil data for AK, may I suggest that you plot Cr vs Ni (linear scale). Then, create a second plot by adjusting the scales to 0-100 for Cr and 0-60 for Ni. A relatively clear relationship is observed for Cr/Ni on the smaller-scaled graph. The resulting Cr/Ni relationship likely describes background minerals, soils and sediment in AK. Now, go back to the full scale graph. Values for Ni >100 and Cr >150 are most likely due to anthropogenic impact or the presence of different natural source minerals.

Visualizing the data as suggested yields a similar insight that our Figure S2 provides by mapping the sites with known contamination from the Alaskan DEC overtop sample sites within the AGDB2 and comparing the proportion of samples with elevated concentrations with and without the mine-impacted sites (with explicit point source contamination) included. These visualizations show that while a fraction of our sites have elevated metals from anthropogenic impacts, the proportion of samples in which metal concentrations likely represent natural levels is greater. The figure below was created using your suggestions, and shows that the proportion of samples with Ni < 100 mg/kg and Cr < 150 mg/kg is larger than those with high concentrations likely reflecting anthropogenic impact. For simplicity, we have chosen to not include this figure as Figure S2 addresses this point in its revised form.

**Reviewer 5:**

Summary

Heavy metals in the environment can result in detrimental effects on the health of the public. Metals have been deposited across the Alaskan Arctic over time, becoming sequestered in permafrost. Melting permafrost due to climate change could result in the release of these metals to the environment and increase exposure to humans and wildlife. This study evaluated soils concentrations of heavy metals across the Alaskan Arctic using publicly available data. Soil heavy metal concentrations in the Alaskan Arctic were generally found to be elevated compared the average concentrations of the contiguous US. This study provides a novel synthesis of soil heavy metal concentrations across the Alaskan Arctic and identifies important gaps for future research efforts. Additionally, this study improves the understanding of health risks associated with possible heavy metal releases from permafrost thaw in the Alaskan Arctic.

**Response to R5:**

Thank you for your careful review of our manuscript. Your suggestions have helped us improve the manuscript’s quality. We have included clarifying statements in our methodology, updated figures, and revised unclear wording in several sections based on your suggestions. Our responses to your individual comments follow.

Major comments

It’s difficult to determine from the methods, but were concentrations of all 5 heavy metal known for any/some/many of the included sampling locations? This could be made clearer in the method. While it may be outside the scope of this study, it would be interesting to look at the relationship among the metals sampled at the same location (if this occurs).

Not all samples contain concentrations of all 5 metals. We have included this note in L151-153 in the revised manuscript:

*“The resulting data table contained 1,151 rows representing individual samples; however, not every sample had a value for the concentration of all 5 metals.”*

Minor comments

Line 47: Change “concentration” to “concentrations”

Done.

Line 76 (88, 113): Be consistent in including the element symbols in the first mention and using the symbol in subsequent mentions

Done.

Line 101: It would be good to focus on health effects that may result from exposure due to release from permafrost, it does not seem relevant to include “respiratory problems from inhalation”

We omitted that portion of the sentence in the revised manuscript (L104-105)

Line 111: The term “permafrost-affected soils” seems strange, maybe use different terminology?

This term is ubiquitous within the scientific literature regarding these systems underlain by permafrost (ground that has been frozen for 2 or more consecutive years, but can still have thaw cycles). While open to using different terminology, there does not seem to be another term that succinctly expresses these soil characteristics.

Line 164 -170 and 216: It would be useful to include how you determined the health standards based on types of exposure. For instance, you reference oral and dermal exposure in line 216 for As. This information should be included in the methods, did you use oral and dermal health standards are each metal used? Were other types for exposure standards used?

Due to the inconsistencies across health standards (described in the revised discussion), we no longer make the comparison to health standards and the changes made have been noted under our response to Reviewer 1.

Line 170-175: Include that these averages are for uncontaminated soils (as explained in line 278)

We have updated the averages we use to compare the concentrations in the Alaskan soils to those from the contiguous United States. See further discussion of this revision in response to Reviewer 1 and Reviewer 4. We have included your suggestion to note that the soils included in these averages were uncontaminated on L176.

Line 198 and 208: Change “sample sources” to “land-cover types”, be sure to keep the terminology consistent

We have changed any reference of “sample sources” to land cover type in the revised manuscript for consistency and to better clarify the meaning of those classifications.

Line 212: Change “source” to “type”

We have moved this figure to the supplement. However, throughout the revised manuscript we have changed “sample source” to “land cover type.”

Line 298-299: Remove “have been found”

This sentence has been omitted from the revised manuscript.

Lines 321-328: It would be good to include some discussion about the depth profile (> 10, 10-30, <30 cm) and how that might relate or be important to transport from melting permafrost

This has been addressed by moving the depth plots from the supplement to the main text and including them in the results/discussion.

Line 334: change “concentration” to “concentrations”

Done.

Fig 1. The pink color of the mine-impacted sites is hard to see, particularly due to the similarity to the pink color of the town/settlements

Fig. 3. This is figure is blurry

Fig. 3. There are some orange dots in the Ni map, are those supposed to be there?

We have resolved image resolution issues and improved map presentation in the revised manuscript.
