# Supplementary material for: Heavy metals in the Arctic: Distribution and enrichment of five metals in Alaskan soils
Source: PLoS One. 2020 Jun 3;15(6):e0233297. doi: 10.1371/journal.pone.0233297 (PMC7269202; doi:10.1371/journal.pone.0233297)
Supplement: Attachment — Submitted filename: AKSoilMetals_ReviewerResponses2.docx [file pone.0233297.s007.docx]

**Reviewer #1:** The comments of 1st reviewer were adequately adressed, and the arguments of authors (where they disagree) are well taken. The paper is in the shape which is suitable for publication; it represents clear added value.

Thank you, your feedback on the original version of the manuscript significantly improved it in its revised form.

**Reviewer #2:** (No Response)

**Reviewer #3:** (No Response)

**Reviewer #4:** The revised manuscript (PONE-D-19-30690R1) is improved due to efforts by the authors to address numerous suggestions from the reviewers. An overreaching difficulty in writing the paper was trying to work with very large ranges and standard deviations (SD) in both the Alaska and the US soil data. I encourage the authors to mention this challenge in the beginning of the Discussion and again in the Conclusions.

Thank you for your additional feedback to our revised manuscript. Please find our responses to your specific comments below. To address your suggestion regarding the large variances in both USGS datasets used in our analyses, we have included the following text:

Discussion (L 253-256): *“An overarching challenge in synthesizing large datasets such as the AGDB2 are the large ranges and variances found in the data, as shown in Table 1. Despite the large variance in the data, the heavy metal concentrations in the soils in the AGDB2 dataset are within the range of those previously reported in other parts of the Arctic.”* (See comment 6 for further discussion of revisions to this paragraph)

Conclusion (L 344-347): *“While the metal concentrations in both USGS datasets include large variances, our synthesis of publicly available data on soil concentrations of As, Cr, Hg, Ni, and Pb in Alaska indicates that metal concentrations in soils in southern and southwestern Alaska often exceed average US soil concentrations by a factor of 2 or more.”*

I have a few suggestions listed below in order of appearance in the text.

1. Line 40 and many others. I do not think that various forms of the word “liberate” (e.g., lines, 40, 64, 69, 72 and several more throughout the text) best explain the process of “release” or “mobilization” of metals from previously frozen soils. I prefer not to see the much overused word “liberate” in the manuscript.

We have opted to use “release” in place of “liberate” throughout the text. Our previous choice of the word liberate was meant to suggest that permafrost thaw can free heavy metals previously bounds in frozen soils, without making a statement about whether they do or do not move through the landscape (as mobilize suggests), as without redox speciation of the metals reported in the ABDB2 we cannot make accurate inferences about the mobility of the metals we report. Of the terms you suggest, we feel that “release” best replaces “liberate.”

2. Line 41. “exacerbate contamination” implies that the soils are already contaminated. Although this is certainly true in a few cases, it does not seem to be the norm nor is it adequately proven. Therefore, “… potentially contaminating food and water” … seems more defendable.

We have revised the text to read (L 40-42):

*“In addition, permafrost thaw may release heavy metals sequestered in previously frozen soils, potentially contaminating food and water resources by increasing the concentration of metals in freshwater, plants, and wildlife.”*

3. Lines 46-57. After listing Items 1), 2) and 3), I prefer to see responses to each to follow in the same order. Therefore, the first sentence after the list would be “We found substantial clustering …”.

We have revised the text to read as follows (L 44-57):

*“We analyzed data of soil concentrations of arsenic, chromium, mercury, nickel, and lead from over 1,000 samples available through the USGS Alaskan Geochemical Database to evaluate 1) the spatial distribution of sampling locations for soil metal analysis, 2) metal concentrations in soils from different land cover types and depths, and 3) the occurrence of soils in Alaska with elevated metal concentrations relative to other soils. We found substantial clustering of sample sites in the southwestern portion of Alaska in discontinuous and sporadic permafrost, while the more populous Interior and continuous permafrost zone in Northern Alaska are severely understudied. Metal concentration varied by land cover type but lacked consistent patterns. Concentrations of chromium, mercury, and lead were higher in soils below 10 cm depth, however these deeper soils are under-sampled. Arsenic, chromium, mercury, nickel and lead concentrations exceeded average values for US soils by one standard deviation or more in 3.7% to 18.7% of the samples in this dataset. Our analysis highlights critical gaps that impede understanding of how heavy metals in thawing permafrost soils may become mobilized and increase exposure risk for Arctic communities.”*

4. Overall, the Abstract, Introduction and Discussion could use some careful editing with a few hours of help from each of the other authors.

All co-authors have read and approved of the text of the manuscript, including the sections mentioned in this comment. To respect the work of the other 4 reviewers who did not have further requests for revisions, we have not significantly changed the text in these sections as you do not provide specific comments beyond those we have already addressed from your comments #1-3 and #5-6.

5. Table 1. Better use of significant figures is essential. Use of more than 3 significant figures seems unacceptable (e.g., 14900.0 [6 sig figs] goes to 14900 [3 sig figs]; 112.73 [5 sig figs] goes to 113 [3 sig figs]).

Pb (mean ± SD) is much more realistic at 21 ± 41, and so on.

Table 1 really prompted my introductory comment about challenges of the large ranges and SDs. Care is certainly required when comparing two data sets with such large ranges and SDs.

To me, range seems better than maximum and minimum (e.g., 2‒720 for Pb).

As an aside, in rethinking the statistics, I probably would have used medians and median

absolute deviations (MAD, as in Smith et al., 2013). No need for the authors to make this change.

We have edited Table 1 so that all values reported have a maximum of 3 significant figures. We have also revised the table to have a column for range instead of separate columns for minimum and maximum.

6. Line 257 and many more. I previously commented “I see too much use of the word ‘comparable’. All data are comparable. The point is whether means for one set of data are statistically different or not from another set of data.”

I still see "comparable" used too often (e.g., lines 257, 259, 264, 267)

We have revised the first paragraph of the discussion to omit the word “comparable” as follows (L 256-271):

*“For example, the median values we report for As, Ni, and Pb of 11.0, 28.3 and 13.0 mg kg^-1^, respectively, fall within the range previously reported in permafrost affected soils from the Lena Delta in Siberia of As ranging from 1.29 to 11.3 mg kg^-1^, Ni from 5.12 to 30.2 mg kg^-1^, and Pb from 2.41 to 13.1 mg kg^-1^ [13]. The interquartile range (IQR) we report for Ni and Pb is the same order of magnitude as the range of concentrations reported by Barker et al. [27] in soil from the Imnavait Creek watershed in Alaska of 13.9 - 23.7 mg kg^-1^ and 4.8 - 13.1 mg kg^-1^, respectively. Halbach et al. [48] measured a suite of metal concentrations in Svalbard, and they report median As, Cr, and Ni concentrations approximately 2 to 4 times lower than the median concentrations reported in this study. Mercury and Pb concentrations from Svalbard [48] of 0.023 to 0.107 mg kg^-1^ and 10.6 to 10.7 mg kg^-1^ are the same order of magnitude as our reported medians of 0.06 mg kg^-1^ and 13.0 mg kg^-1^. Similar levels of soil Hg have been reported from numerous sites across the Arctic, including the Canadian High Arctic [49], the Taimyr Peninsula [50], Lena Delta [13] in Siberia, Alaska [12], and Sweden [51]. The median and IQR of Hg concentrations reported here are in agreement with previous work which finds that Hg in Arctic soils tends to range from below detection (~0.01 mg kg ^-1^) to below 0.1 mg kg^-1^.”*

7. I think the authors make their point and thus the Conclusion seems to work well.

Thank you, your thorough feedback on the original version of the manuscript significantly strengthened it in its revised form.

**Reviewer #5:** (No Response)
